# Risk Factors for Recurrent Exacerbations in the General-Practitioner-Based Swiss Chronic Obstructive Pulmonary Disease (COPD) Cohort

**DOI:** 10.3390/jcm12206695

**Published:** 2023-10-23

**Authors:** Nebal S. Abu Hussein, Stephanie Giezendanner, Pascal Urwyler, Pierre-Olivier Bridevaux, Prashant N. Chhajed, Thomas Geiser, Ladina Joos Zellweger, Malcolm Kohler, David Miedinger, Zahra Pasha, Robert Thurnheer, Christophe von Garnier, Joerg D. Leuppi

**Affiliations:** 1 University Institute of Internal Medicine, Cantonal Hospital Baselland, 4031 Liestal, Switzerland; nebal.abuhussein@yale.edu (N.S.A.H.); ste.giezendanner@protonmail.com (S.G.); pchhajed@gmail.com (P.N.C.); david.miedinger@unibas.ch (D.M.); zahra.pasha@ksbl.ch (Z.P.); 2 Medical Faculty, University of Basel, 4001 Basel, Switzerland; 3 Department of Pulmonary Medicine, Inselspital, Bern University Hospital, 3012 Bern, Switzerland; thomas.geiser@insel.ch; 4 Department for BioMedical Research, University of Bern, 3012 Bern, Switzerland; 5 Pulmonary, Critical Care & Sleep Medicine, Yale School of Medicine, New Haven, CT 06510, USA; 6 University Hospital Basel, 4031 Basel, Switzerland; pascal.urwyler@usb.ch; 7 Service de Pneumologie, Hôpital du Valais, 1950 Sion, Switzerland; pierre-olivier.bridevaux@hopitalvs.ch; 8 St. Claraspital, 4058 Basel, Switzerland; ladina.jooszellweger@claraspital.ch; 9 University Hospital Zurich, 8091 Zurich, Switzerland; malcolm.kohler@usz.ch; 10 Kantonsspital Muensterlingen, 8596 Münsterlingen, Switzerland; robert.thurnheer@stgag.ch; 11 Division of Pulmonology, Department of Medicine, CHUV, University Hospital Lausanne, University of Lausanne, 1011 Lausanne, Switzerland; christophe.von-garnier@chuv.ch

**Keywords:** COPD, exacerbation, primary health care, risk factors, prediction, recurrent exacerbations

## Abstract

Background: Patients with chronic obstructive pulmonary disease (COPD) often suffer from acute exacerbations. Our objective was to describe recurrent exacerbations in a GP-based Swiss COPD cohort and develop a statistical model for predicting exacerbation. Methods: COPD cohort demographic and medical data were recorded for 24 months, by means of a questionnaire—based COPD cohort. The data were split into training (75%) and validation (25%) datasets. A negative binomial regression model was developed using the training dataset to predict the exacerbation rate within 1 year. An exacerbation prediction model was developed, and its overall performance was validated. A nomogram was created to facilitate the clinical use of the model. Results: Of the 229 COPD patients analyzed, 77% of the patients did not experience exacerbation during the follow-up. The best subset in the training dataset revealed that lower forced expiratory volume, high scores on the MRC dyspnea scale, exacerbation history, and being on a combination therapy of LABA + ICS (long-acting beta-agonists + Inhaled Corticosteroids) or LAMA + LABA (Long-acting muscarinic receptor antagonists + long-acting beta-agonists) at baseline were associated with a higher rate of exacerbation. When validated, the area-under-curve (AUC) value was 0.75 for one or more exacerbations. The calibration was accurate (0.34 predicted exacerbations vs 0.28 observed exacerbations). Conclusion: Nomograms built from these models can assist clinicians in the decision-making process of COPD care.

## 1. Introduction

Chronic obstructive pulmonary disease (COPD) is a highly prevalent disease. According to the World Health Organization, it is currently the third leading cause of death worldwide [1,2]. An acute exacerbation of COPD adds to the disease burden and increases the morbidity of COPD. Furthermore, it leads to lung function decline, the deterioration of quality of life, and increased mortality. Frequent exacerbations of COPD can lead to an emergency room visit followed by hospitalization, thus leading to a heavy economic and social burden. COPD exacerbations account for 50–75% of the total cost of COPD healthcare management [3]. Severe acute COPD exacerbations have a negative impact on patients’ prognosis regarding disease prognosis, quality of life, and mortality [4]. According to the Global Initiative on Chronic Obstructive Lung Disease (GOLD), exacerbation prevention and reducing the frequency and severity of exacerbations are the main goals of the management of COPD besides improving quality of life and slowing down disease progression [4]. A better understanding of disease progression and distinguishing symptoms or factors that can aid in predicting exacerbation would help physicians to recognize exacerbations on time and treat their patients efficiently. Management calculation tools such as DOSE, APACHE, and BODE are common in medicine, especially in chronic disease management [5,6,7]. The existence of management prediction tools can help physicians by allowing them to manage their patients and predict their disease development and progression. There are few models that specifically predict COPD exacerbation [8,9,10,11], and most of these models require hospitalization or complicated tools like CT scans or questionnaires. Moreover, most of these models were developed in a hospital setting and not in primary care settings where most COPD patients are managed, making them too unrealistic to be implemented in real-life settings. The Swiss COPD cohort is an ongoing cohort monitored since 2009 with the aim of improving the management and quality of life of COPD patients in primary care settings. The data collected within this cohort have included demographic data, treatment, and exacerbation data [12,13]. The aim of this study was to evaluate the best COPD exacerbation predictors in our Swiss primary-care-based COPD cohort and construct a simple tool with which to model the annual exacerbation rate.

## 2. Materials and Methods

### 2.1. Study Population and Study Design

We analyzed the data from the ongoing Swiss COPD cohort from 2014 and 2022 [13]. For this ongoing questionnaire-based observational cohort study, general practitioners (GPs) from all over Switzerland were invited to participate in the cohort study. In total, 139 GPs from 23 Swiss cantons agreed to participate. Each physician recruited 1 to 20 patients with presumed COPD and performed follow-ups over a total period of 24 months or longer. Written informed consent was obtained from our patients. All COPD patients treated in the GPs’ practice were allowed to participate in the study. The inclusion/exclusion criteria for the cohort as listed in the original study protocol published at clinicaltrials.gov are as follows:

Inclusion Criteria:

- Tiffenau (FEV17FVC) < 70 without reversibility (increase in FEV1 after inhalation of a bronchodilator <200 mL and <12%);
- Age: >40 years;
- Both genders;
- Smokers or ex-smokers with at least 20 pack years;
- Informed consent.

Exclusion Criteria:

- <40 years of age
- Tiffenau (FEV17FVC) > 70.

All ethical committees of the participating Swiss cantons gave their ethical approval for the study in 2006. In this questionnaire-based cohort, the doctors saw the patients in at least 6-month intervals.

Data collection included demographic data, physical examination information, spirometric parameters, symptoms (sputum production, dyspnea), comorbidities, medical treatment history, and exacerbation history. Age, gender, height, weight, body mass index, and current smoking status were recorded at the baseline visit and updated in the following visits. Information about changes in medication, recent hospitalizations, and exacerbations since the last visit was documented. Exacerbation was defined as worsening of clinical symptoms leading to a change in treatment.

Spirometry (EasyOneTM, ndd Medizinitechnik AG, Zurich, Switzerland) was performed according to the guidelines of the American Thoracic Society and European Respiratory Society (ERS ATS) [14] and as described in our previous publications [12,13,15]. All participating physicians were instructed on the usage of the spirometer and the administration of the test. Anonymized data were entered into an online database (RDE Light) either by the physicians or by the study team after receiving the collected data questionnaires via facsimile.

### 2.2. Assessment of Severity of COPD

The severity of COPD was assessed using spirometric data provided by the GPs and interpreted according to GOLD criteria [16]. All patients were classified into risk groups A to D according to the revised GOLD guidelines 2011 [2].

### 2.3. COPD Assessment Test (CAT)

CAT is a short health status questionnaire developed to provide a simple tool for assessing the impact of COPD. The questionnaire contains 8 items, each presented as a semantic 6-point differential scale, providing a total score ranging from 0–40. The CAT covers daily symptoms, such as cough, phlegm, and chest tightness, as well as other manifestations of COPD like breathlessness when ascending hills/stairs, activity limitations at home, decreased confidence in leaving home, and limited sleep and energy [17].

### 2.4. Modified Medical Research Council Dyspnea Scale

The mMRC dyspnea scale is a modified version of the original MRC dyspnea scale developed by Fletcher in 1952. It contains more simplified statements and is based on 5 stages of exertional dyspnea ranging from 0 to 4 [18].

### 2.5. Statistical Analysis

Continuous variables were given as means and standard deviations, and categorical variables were presented as absolute and relative frequencies. Student’s two-sample *t*-test was used to analyze continuous variables across validation and training datasets, and Pearson’s χ^2^ test was used for the comparison of categorical variables across validation and training datasets (see). All statistical and machine-learning analyses were performed using R [19].

### 2.6. Description of Recurrent Event Data

Nonparametric mean cumulative function (MCF) estimates are widely utilized in exploring the trends of recurrent event data. Thus, for the visualization of recurrent exacerbations, we estimated the overall sample mean cumulative function (MCF) [20,21,22], which is the average number of cumulative exacerbations experienced by an individual in the study at each point in time, since the start of a follow-up using the “mcf” function from the R package “reReg” [23]. For variance estimation, we used the Lawless and Nadeau method [21].

The MCF estimates were computed based on each unique time point of the sample data. By default, the size of the risk set was adjusted over time based on the at-risk indicators, resulting in the Nelson–Aalen nonparametric estimator. We extracted the overall MCF at the 1st- and 2nd-year follow-ups. Further, we produced event plots to show each individuals’ event history across time using the function “plotEvents” from the R package “reReg”.

### 2.7. Analysis of Recurrent Event Data Using Negative Binomial Regression

The primary outcome was the exacerbation rate. The effect of risk factors was evaluated using the negative binomial regression analysis implemented with the “glm.nb” function of the R package “MASS”. To account for the different lengths of follow-ups between patients, we included an offset term denoting the logarithm of the duration of a follow-up. Univariable and multivariable regression analyses were performed to investigate independent risk factors that might be associated with the risk of exacerbations. The data were split into a training dataset consisting of 75% of the data and a validation dataset consisting of 25% of the data. One of the goals of supervised learning is to build a model that performs well using new data. If one has new data, it is a good idea to see how well one’s model performs when using them. The problem is that new data might not be available, but one can simulate this experience with a procedure like splitting a collection of data into a training dataset (75% of a collection of data split using random sampling without replacement) and a testing dataset (25% of the remaining data). This is a model validation process that allows one to simulate how a model would perform with new data. We prespecified possible predictors for the multivariable model based on clinical relevance and availability of predictors in all datasets. Predictors included the occurrence of exacerbations over the previous year or at baseline, baseline age, sex, smoking status, post-bronchodilator FEV1 value (% of predicted), mMRC Dyspnea Scale score, body-mass index, the use of COPD medications, reception of domiciliary oxygen therapy at baseline, and comorbidities such as asthma, coronary heart disease, hypertension, diabetes, and cancer. COPD medications were defined as long-acting muscarinic receptor antagonists, short- and long-acting β2 agonists, and inhaled corticosteroids as well as their combinations. If the relative frequency of a variable was below 10%, it was excluded from the multivariable analysis. A multivariable best subset of the predictors for the exacerbation rate was selected using Akaike’s information criterion (AIC) via the training dataset and a stepwise backward algorithm. The IRR and 95% CI for each variable were calculated.

### 2.8. Assessment of Performance

The best subset model was validated in the validation dataset. We examined model calibration—the degree to which predicted and actual risks or rates of exacerbations aligned—and discrimination (the extent to which the model separated individuals with different risks). Discrimination was assessed by calculating receiver operating characteristic (ROC) curves and the area under the curve (AUC). Calibration was assessed by comparing the predicted and observed exacerbation rates evaluating calibration plots and via calculating Brier scores (i.e., the mean squared error of forecast).

### 2.9. Nomogram

A nomogram for predicting the annual exacerbation rate was developed based on the multivariable best subset in the training dataset. We used the “rms” package of the R software (https://www.r-project.org/, accessed on 11 October 2023) to develop nomograms in order to visualize our predictive model graphically.

## 3. Results

### 3.1. Demographic and Baseline Data

A total of 139 GPs from Switzerland agreed to participate in this study and recruited 328 patients between 2014 and 2022. In total, 299 of the subjects suffered from COPD according to the GOLD criteria (FEV1/FVC ratio under 0.7). A total of 43 cases were excluded because they did not attend a follow-up visit. The final analysis was performed using the complete set of data available for 256 patients recruited by 21 centers. The descriptive baseline data are shown in Table 1.

### 3.2. Recurrent Event Process

For the 256 patients, 98 exacerbations occurred during a median follow-up time of 2 years. 

**Figure 1:**
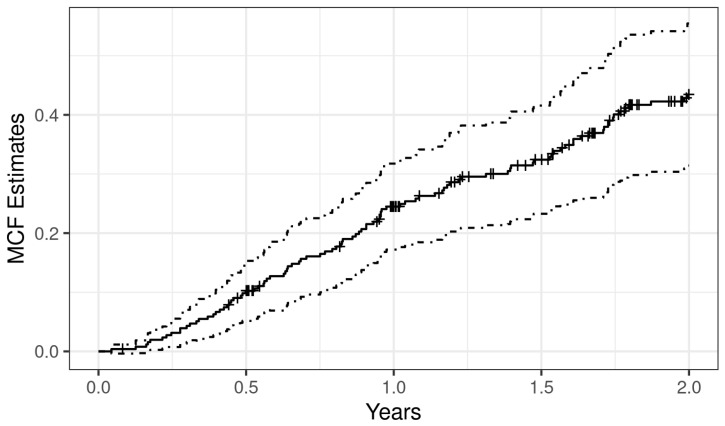
Non-parametric overall mean cumulative function estimate of exacerbation. The *x*-axis depicts the time since study entry, and the *y*-axis represents the average number of exacerbations an individual experienced during their follow-up.

Figure 1 shows the mean cumulative function (MCF) of exacerbation for all patients. The average number of recurrent exacerbations per subject was estimated to be 0.38. At one year, the MCF was 0.24 (95%CI = 0.17–0.32), which means that a patient experienced, on average, 0.24 exacerbations over the first year of follow-up in this study.

**Figure 2:**
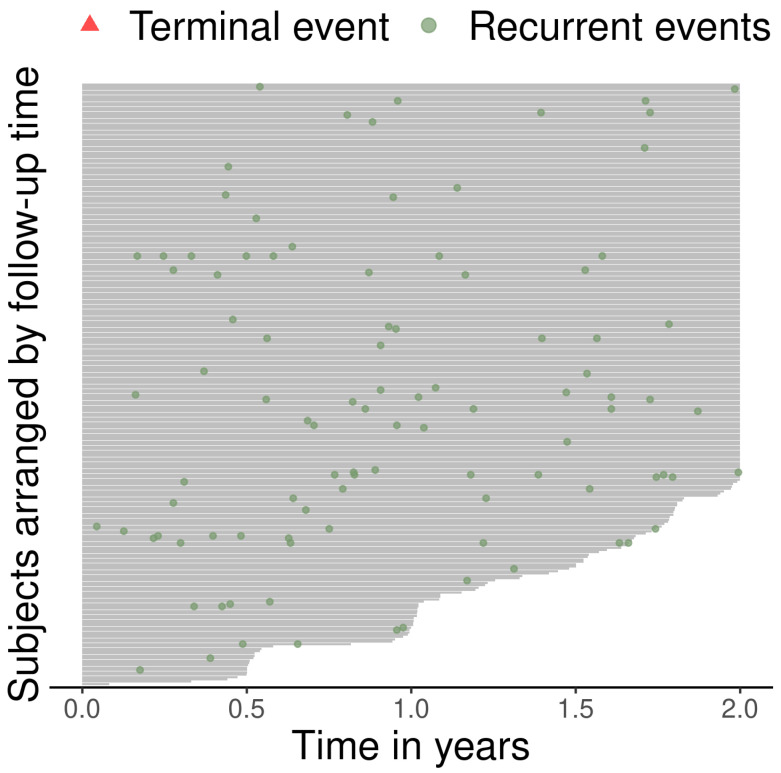
Event history of the data. The length of the lines indicates the length of follow-up, and the green dots indicate the exacerbations. There was no terminal events over the observation period.

The follow-up and event history of each individual is visualized in Figure 2. Three quarters of the patients (*n* = 193) had no exacerbations during their follow-ups (see Figure 2 for information on the number of recurrences per individual).

### 3.3. Factors Associated with Recurrent Exacerbations

A univariate analysis was performed to assess factors associated with the risk of recurrent exacerbations for the entire training dataset. This analysis led to the following results: Of the factors listed in table two, LABA, LAMA, and ICS followed by exacerbation history of the past year had the highest association with exacerbation. On the other hand, the combination of LABA and ICS had the least significant association with exacerbation in this training dataset. Surprisingly, patients with SABA-only inhalers had a high association with exacerbation (IRR: 1.22).

Using the above factors from Table 2, we built the best subset model using the multivariable negative bi-nominal regression model. This model included only five factors: FEV1, mMRC dyspnea scale, combination therapy with LABA + ICS, combination therapy LABA/LAMA/ICS, and exacerbation history. This information is depicted in Table 3.

The discrimination and clinical utility of the NBR model for predicting exacerbation rate.

**Figure 3:**
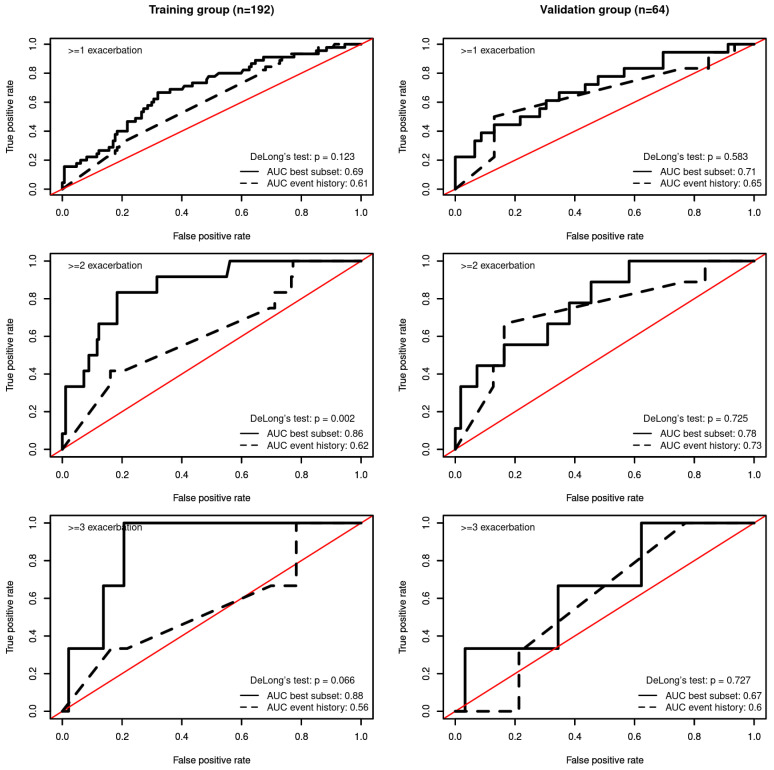
Receiver operating characteristic curve corresponding to the negative binomial regression model. (**Left**) Area under the curve (AUC) for predicting the occurrence of ≥1 (**top**), ≥2 (**middle**), and ≥3 (**bottom**) exacerbations in the training group. (**Right**) AUC for predicting the occurrence of ≥1 (**top**), ≥2 (**middle**), and ≥3 (**bottom**) exacerbations in the validation group.

The area under the curve (AUC) was above 0.7 for the validation group with respect to predicting ≥ 1 and ≥2 exacerbations (see Figure 3 and Table 4).

**Figure 4:**
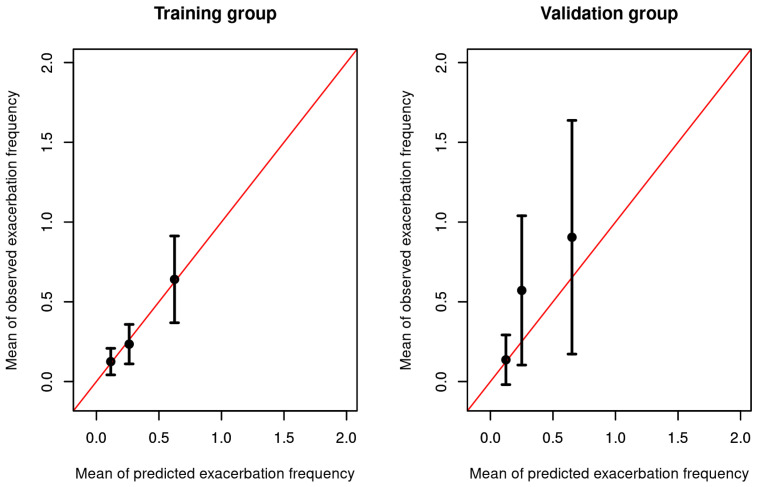
Calibration plots of the training dataset on the left and of the validation dataset on the right. Both samples used for validation were divided into 3 groups according to their predicted risk with bin sizes of equal length. For each group, the mean predicted risk and the mean observed cases are shown on the *X* and *Y* axes, respectively. Bars indicate 95% confidence intervals of the mean.

After stratifying each model into three risk groups according to their predicted incidence rate ratios based on the best subset model, the predicted number of exacerbations was within the 95% CI of the observed exacerbation rate in all risk groups for the training and the validation datasets (see Figure 4). Regarding calibration, the best subset model predicted an average of 0.34 exacerbations in the validation dataset (considering the observation time), while, in reality, there was an average of 0.53 observed exacerbations. The Brier score was 0.15 for the training dataset and 0.08 for the validation dataset.

Compared with existing practice, which relies exclusively on the previous history of exacerbation to predict the future risk of exacerbation, the best subset model was better at predicting ≥ 2 exacerbations for the training dataset (AUCbest subset = 0.86 vs. AUCevent history = 0.62; De Long’s Test *p* < 0.002).

### 3.4. Nomogram

**Figure 5:**
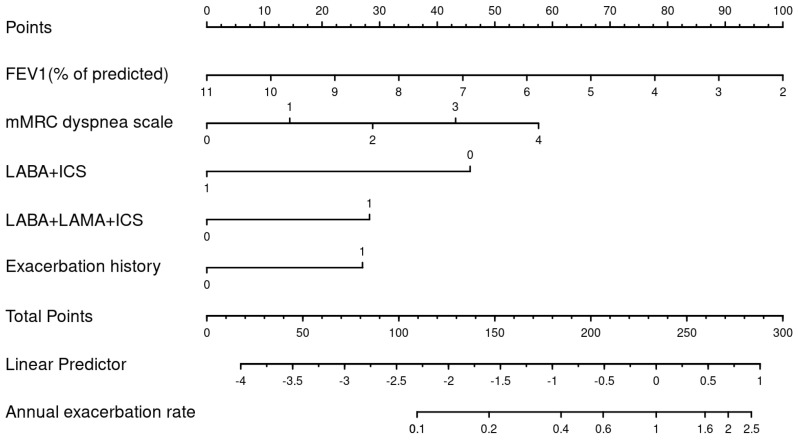
A nomogram for predicting annual exacerbation rates among patients with COPD in primary care.

Using the best subset model, we developed a nomogram, which could be used to manually obtain predicted exacerbation rates from the regression model within one year (see Figure 5). The five factors associated with exacerbation included in the nomogram were triple therapy with LABA/LAMA/ICS, exacerbation history during the past year, baseline FEV1 value, mMRC dyspnea scale score, and treatment with LABA/ICS.

**Figure 6:**
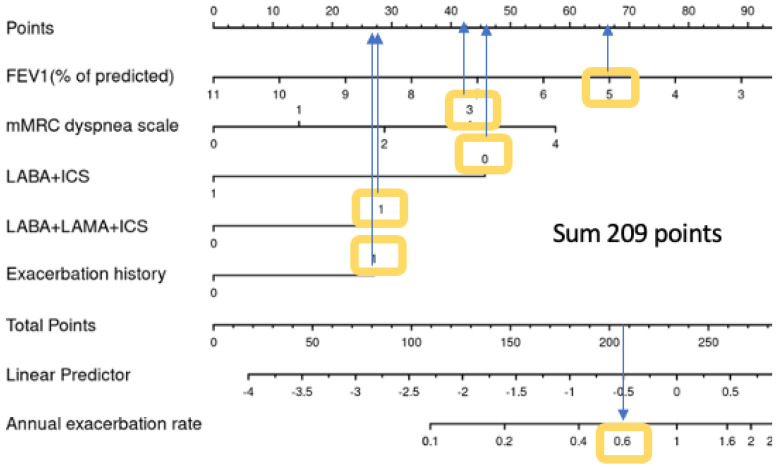
Nomogram with a case example.

Figure 6 shows a real-life example of how to use the nomogram to calculate the exacerbation risk for a patient during a routine visit: A 55-year-old male COPD patient who is still a smoker, has experienced an exacerbation during the last year, and is undergoing LABA/LAMA/ICS as an inhalative therapy reports shortness of breath after a few minutes of walking on level ground (mMRC3). His physical examination shows that his lung function test results reveal an FEV1 value of 50%, a BMI of 25 kg/m^2^, and, currently, no signs of exacerbation. Using the nomogram, the patient has a sum of 209 points; according to the linear predictor, his exacerbation risk will be 0.6 for the next year.

## 4. Discussion

Exacerbation is a global burden in the field of COPD care, and its prevention is one of the aims and challenges of primary care. This study has aimed to fill the niche of managing COPD patients using predictors. Perez et al. state that primary care physicians face significant challenges in managing and caring for 80% of COPD patients [24]. Some of the biggest challenges include being able to effectively lower the exacerbation rate and thus the hospitalization rate [25]. The aim of our study was to investigate prediction factors for exacerbation in a Swiss general-practitioner-based cohort and to build a prediction model for use in primary care.

Having assessed previous models of exacerbation prediction, this study decided it was important to include factors that are more accessible for GPs and more plausible and primary-care-oriented, as most COPD patients are treated in primary care.

In turn, this enables the treating physician to make changes to their patient’s treatment, allowing for better-quality patient care using a long-term perspective of the condition.

LABA/LAMA/ICS closely followed by exacerbation were the strongest predictors for future exacerbation according to our uni- and multivariate analyses. These findings align with those of other studies, which have shown that previous exacerbation is the strongest predictor for future exacerbations [7,26]. Furthermore, the importance of exacerbation as a factor has been noted, as it has a significant effect on a patient’s prognosis and disease progress. The fact that triple therapy is the highest predictor shows that GPs react to frequent exacerbation according to the corresponding guidelines. We also observed such a practice carried out by the GPs who successfully treated COPD patients for exacerbations, demonstrating best practice as per the guidelines.

In contrast to many other studies, such as the ECLIPSE study, self-reported dyspnea was a very strong predictor and significant in both uni- and multivariable analysis. Furthermore, we observed an association with FEV1 value and with combination therapy with LABA and ICS; the latter therapy was applied to 14% of all patients (12.5% of patients in the training set and 18.75% of the validation data set), and it is also a therapeutic option for patients within group D with frequent exacerbations.

Surprisingly, smoking could not be associated with exacerbation in either the univariable or multivariable analyses. Soler-Cataluna et al. reported similar observations for their cohort as well as in other studies [27,28,29]. For several other cohorts, smoking was a significant predictor for exacerbation. The differences in observations may be due to varying populations and analysis methods [26].

Studies such as those conducted by Marshal et al., Kim et al., and Rahman et al. [30,31,32,33,34] have confirmed the importance of exacerbation by showing its association with high mortality rates after hospital admission. However, it must be pointed out that better management can be achieved through exacerbation control, where such outcomes can be avoided and better quality of life for patients can be achieved. Although the development of prediction models is generally not a new topic in medicine and in the management of chronically ill patients in general and COPD in particular [7,35,36,37,38], our model presents GPs with parameters that are easier to incorporate into patient care. Sin et al. developed the ACCEPT tool to predict exacerbation, which was updated and published in 2022 [11,39]. In contrast to the ACCEPT tool for the prediction of exacerbation, our prediction tool uses fewer, easier-to-obtain variables in primary care. Furthermore, we included all patients treated in primary care and patients without any exacerbations, that were excluded in the ACCEPT cohorts. Our model showed a superior AUC curve in terms of predicting two or more exacerbations.

To the best of our knowledge, there is still an existing niche in the field for a model that can help predict exacerbations, using a nomogram, for primary care patients. To emphasize the lack of such research, it should be noted that we could find only one published study from Bertens and colleagues; this study predicted exacerbation and suggested that previous exacerbations, predicted FEV1 value, pack years of smoking, and history of vascular disease were good predictors of exacerbation [40].

With the help of our nomogram, which encompasses five easily obtainable parameters, a GP can calculate the probability of exacerbation and decide whether to change a treatment or proceed further with the same management strategy. By using clear parameters of treatment, a GP can manage each patient individually according to his/her risk profile. Many studies have shown that the severity of exacerbations increases over time [41,42]. We believe that an easy-to-obtain calculation model can help fill the niche of more effective treatment strategies, enabling better patient care (and communication) in the treatment for COPD patients.

The fact that our study is a primary-care study is one of its main strengths since most COPD patients are treated and managed in primary care, and this is very important in terms of result interpretation. Furthermore, our study population is similar to populations analyzed in several primary care studies, indicating the generalizability of this study [28,43,44]. Another main strength of our study is that we followed the patients for at least two years, which allowed us to observe the changes in exacerbation for a sufficient amount of time.

Despite the promising findings generated by our study, our study has some limitations, such as its overall relatively small patient population. Within GOLD stages I and IV in particular, we believe that with more patients, we could have received higher-quality data, especially if we had a significantly higher number of patients in each of the GOLD stages. As a cohort study that has been ongoing for a long time, we have a considerable number of patients lost during follow-ups; this could have affected our results negatively. Another limitation would be the unequal presentation of gender in our cohort, as most of our cohort consisted of males. An additional limitation was that we did not have an external validation cohort for our developed model. This study notes this lack as an important element to control in the model via an external validation cohort and looks to fulfil this with future work. Lastly, we would like to add that this study was only an observational cohort study; therefore, judgement on the GPs’ therapeutic decisions was withheld.

## 5. Conclusions

In conclusion, our study confirms that a history of exacerbation is the most important predictor for a future exacerbation, alongside severe symptoms like dyspnea and sputum, which has also been confirmed in the findings of several studies.

This offers the opportunity to provide a more effective resource for exacerbation measurement in the years following consultation, enabling a more efficient, medically accurate means of disease control/exacerbation measurement. Despite these important strengths, it is important that the prediction model is validated using a different external cohort. Another recommendation would be to develop a randomized clinical trial to test the model in a clinical setting.

## Figures and Tables

**Table 1:** Comparison of descriptive characteristics between the training dataset and the validation dataset.

General Characteristics	All Patients	Training Dataset	Validation Dataset	*p* Value
N total	256	192	64	
Age (years), Mean (SD)	67.62 (10.17)	67.86 (10.25)	66.88 (9.95)	0.496
BMI (kg/m^2^), Mean (SD)	26.93 (6.07)	26.88 (6.35)	27.09 (5.21)	0.786
Male sex	162 (63.28%)	120 (62.5%)	42 (65.62%)	0.765
Current smoker	125 (48.83%)	92 (47.92%)	33 (51.56%)	0.718
Lung function				
FEV1 (% of predicted), Mean (SD)	59.57 (18.15)	59.79 (18.6)	58.91 (16.87)	0.725
FVC (% of predicted), Mean (SD)	84.34 (22.51)	84.83 (23.74)	82.88 (18.43)	0.496
FEV1/FVC, Mean (SD)	56.09 (9.75)	55.93 (9.65)	56.57 (10.1)	0.658
GOLD 1	38 (14.84%)	31 (16.15%)	7 (10.94%)	0.708
GOLD 2	139 (54.3%)	101 (52.6%)	38 (59.38%)	
GOLD 3	68 (26.56%)	52 (27.08%)	16 (25%)	
GOLD 4	11 (4.3%)	8 (4.17%)	3 (4.69%)	
Symptoms				
mMRC dyspnea scale 0	22 (8.59%)	15 (7.81%)	7 (10.94%)	0.344
mMRC dyspnea scale 1	103 (40.23%)	73 (38.02%)	30 (46.88%)	
mMRC dyspnea scale 2	89 (34.77%)	73 (38.02%)	16 (25%)	
mMRC dyspnea scale 3	35 (13.67%)	25 (13.02%)	10 (15.62%)	
mMRC dyspnea scale 4	7 (2.73%)	6 (3.12%)	1 (1.56%)	
COPD treatment				
No LAMA, LABA, or ICS	28 (10.94%)	22 (11.46%)	6 (9.38%)	0.817
On short-acting bronchodilators (SABA) only	77 (30.08%)	60 (31.25%)	17 (26.56%)	0.582
On long-acting muscarinic antagonists (LAMA)	43 (16.8%)	32 (16.67%)	11 (17.19%)	1
On long-acting ß2-agonists (LABA) only	4 (1.56%)	3 (1.56%)	1 (1.56%)	1
On inhaled corticosteroids (ICS) only	5 (1.95%)	3 (1.56%)	2 (3.12%)	0.794
Inhaled combination therapy (LABA+ICS)	36 (14.06%)	24 (12.5%)	12 (18.75%)	0.299
Combination therapy (LABA+LAMA)	79 (30.86%)	63 (32.81%)	16 (25%)	0.31
LABA + LAMA + ICS	63 (24.61%)	46 (23.96%)	17 (26.56%)	0.802
On systemic corticosteroids	7 (2.73%)	6 (3.12%)	1 (1.56%)	0.825
O2 therapy previous year	17 (6.64%)	10 (5.21%)	7 (10.94%)	0.192
Physical activity				
Exercise (at least twice a week)	84 (32.81%)	60 (31.25%)	24 (37.5%)	0.442
Pulmonary rehabilitation	15 (5.86%)	13 (6.77%)	2 (3.12%)	0.442
Comorbidities				
Asthma	31 (12.11%)	21 (10.94%)	10 (15.62%)	0.439
Hypertension	128 (50%)	98 (51.04%)	30 (46.88%)	0.665
Coronary heart disease	31 (12.11%)	24 (12.5%)	7 (10.94%)	0.912
Heart failure	13 (5.08%)	9 (4.69%)	4 (6.25%)	0.869
Peripheral artery disease	21 (8.2%)	18 (9.38%)	3 (4.69%)	0.357
Cerebrovascular Insult	8 (3.12%)	5 (2.6%)	3 (4.69%)	0.678
Diabetes	31 (12.11%)	23 (11.98%)	8 (12.5%)	1
Cancer	10 (3.91%)	9 (4.69%)	1 (1.56%)	0.456
Exacerbation history over the past year	66 (25.78%)	49 (25.52%)	17 (26.56%)	1
Outcome				
Exacerbation count: 0	193 (75.39%)	147 (76.56%)	46 (71.88%)	0.123
Exacerbation count: 1	42 (16.41%)	33 (17.19%)	9 (14.06%)	
Exacerbation count: 2	15 (5.86%)	9 (4.69%)	6 (9.38%)	
Exacerbation count: 3	3 (1.17%)	1 (0.52%)	2 (3.12%)	
Exacerbation count: 5	2 (0.78%)	2 (1.04%)	0 (0%)	
Exacerbation count: 7	1 (0.39%)	0 (0%)	1 (1.56%)	
Follow-up time (years), Mean (SD)	193 (75.39%)	147 (76.56%)	46 (71.88%)	0.123

Demographic characteristics are shown for the imputed dataset. Missing values were observed for the following variables: Age: 3, Sex: 2, BMI: 4, Smoking: 3; they were imputed with knn. Therapy also had missing values, namely, SABA: 1, LABA: 1, LAMA: 1, ICS:1, LABA/ICS = 2, and LABA/LAMA = 5; these were not imputed but replaced with 0.

**Table 2:** Factors associated with exacerbation rate according to a univariable analysis conducted on the training dataset (*n* = 172).

Factors	IRR	95% CI Lower	95% CI Upper	*p*-Value
LABA/LAMA/ICS	2.5	1.34	4.67	0.004
Exacerbation history over the past year	2.06	1.08	3.93	0.027
mMRC dyspnea scale (per score)	1.48	1.07	2.07	0.022
Asthma	1.36	0.53	3.37	0.517
Age (per 10 years)	1.22	0.88	1.71	0.208
On SABA only	1.21	0.62	2.32	0.574
BMI (per 10 kg/m^2^)	0.88	0.53	1.43	0.614
Hypertension	0.88	0.48	1.63	0.688
Diabetes	0.86	0.29	2.3	0.773
On LAMA only	0.83	0.34	1.92	0.666
Current smoker at baseline	0.82	0.44	1.51	0.522
On LABA/LAMA	0.78	0.4	1.52	0.479
Male vs female sex	0.76	0.41	1.42	0.381
Coronary heart disease	0.75	0.25	2	0.582
Baseline FEV1 (per 10% of predicted)	0.74	0.62	0.88	0.001
On LABA + ICS	0.36	0.08	1.15	0.116

**Table 3:** Best subset model according to multivariable negative binomial regression conducted on the training dataset (*n* = 172).

	IRR	2.50%	97.50%	*p*-Value
Baseline FEV1 (per 10% of predicted)	0.81	0.68	0.97	0.027
mMRC dyspnea scale (per score)	1.3	0.94	1.81	0.123
LABA/ICS	0.43	0.1	1.33	0.183
LABA/LAMA/ICS	1.69	0.9	3.14	0.102
Exacerbation history in the past year	1.65	0.89	3.02	0.108

**Table 4:** AUC for predicting exacerbation frequency in the training and the validation data.

Number of Exacerbations	AUC	AUC 95% Lower Limit	AUC 95% Lower Limit	Sensitivity at Best Threshold *	Specificity at Best Threshold *
Training					
≥1	0.69	0.60	0.78	0.67	0.68
≥2	0.86	0.76	0.96	0.83	0.82
≥3	0.88	0.77	0.99	1.00	0.79
Validation					
≥1	0.71	0.56	0.85	0.67	0.65
≥2	0.78	0.62	0.93	0.89	0.55
≥3	0.67	0.32	1.00	1.00	0.38

* Youden’s J statistic (Youden, 1950); the optimal cut-off is the threshold that maximizes the distance to the identity (diagonal) line.

## Data Availability

The datasets used and analyzed in this study are available from the corresponding authors upon reasonable request.

## Abbreviations

COPD	chronic obstructive pulmonary disease
LABA	long-acting beta-agonists
LAMA	long-acting muscarinic receptor antagonists
ICS	inhaled corticosteroids
SABA	short-acting bronchodilators
mMRC	Modified Medical Research Council Dyspnea Scale
